# Retroperitoneal Inflammatory Myofibroblastic Tumor: Case Report and Immunohistochemistry Study

**DOI:** 10.4021/wjon2010.03.192w

**Published:** 2010-04-30

**Authors:** Sonia Ziadi, Mounir Trimeche, Sarra Mestiri, Wafa Joma, Moncef Mokni, Rached Lataif, Badreddine Sriha, Sadok Korbi

**Affiliations:** a Departments of Pathology, CHU Farhat-Hached, Tunisia; b Departments of Surgery, CHU Farhat-Hached, Tunisia

**Keywords:** Retroperitoneum, Inflammatory myofibroblastic tumor, P53

## Abstract

Inflammatory myofibroblastic tumors (IMT) are a rare clinicopathological entity of yet unknown etiology and those located retroperitoneally are even rarer. Clinical outcome is unpredictable and complete surgical resection of the tumor remains the principal treatment. We report the case of a 41-year old man presented with abdominal pain. An abdominal magnetic resonance imaging scan revealed a retroperitoneal tumor located between the pancreas, stomach small curvature and big vessels. A laparotomy with biopsy was performed because the tumor was not amenable to surgical resection. Histopathological examination concluded to an IMT with overexpression of protein p53. Epstein-Barr virus and Human Herpesvirus-8 investigation was negative. Postoperative outcome was unfavorable.

## Introduction

Inflammatory myofibroblastic tumor (IMT), also known as inflammatory pseudotumor, is an uncommon tumor, characterized by a controversial etiology, various histopathologic features and an unpredictable biological behavior. They can be found virtually at any anatomic site, with a predilection for the lung, the genito-urinary tract and the mesentery [1]. Retroperitoneal location has been rarely reported.

## Case Report

**Figure 1:**
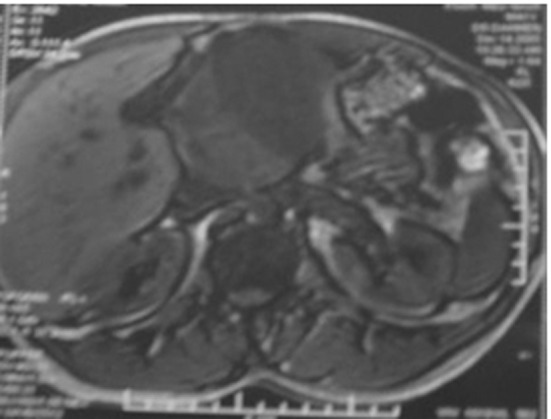
Magnetic resonance imaging: large retroperitoneal mass compressing the liver.

A 41-year old man presented with history of epigastric pain, abdominal lump of 2 years duration. Physical examination revealed a deeply located mass at abdominal palpation. There were no evident foci of infection. The leukocyte count was normal. A hypochromic microcytic anemia was found. The platelet count was normal. Computerized tomography (CT) and magnetic resonance imaging (MRI) revealed a large mass with well-defined borders, located between the stomach small curvature, pancreas and the liver (Fig. 1), without calcification or contrast enhancement, raising the suspicion of a mesenchymal tumor. A laparotomy was performed. The mass was retro-gastric, adherent to the pancreas and the portal veins. The tumor was partially resected. No palliative treatment was administrated. Postoperatively, the patient presented infectious complications and died 3 months later after the initial diagnosis.

**Figure 2:**
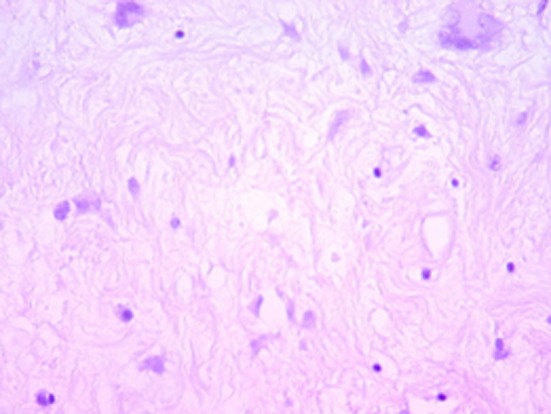
Hematoxylin-eosin x 200: bland fusiform and multinucleate cells admixed with inflammatory cells, mainly lymphocytes within a myxoid stroma.

**Figure 3:**
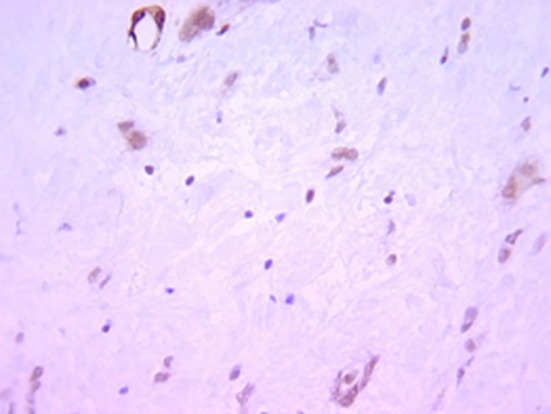
Immunihistochemistry x 200: the tumor cells express smooth muscle actin.

**Figure 4:**
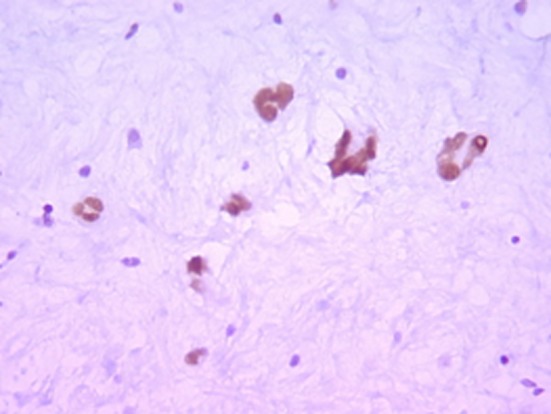
Immunihistochemistry x 200: nuclear expression of p53 by tumor cells.

Histologically, the tumor was well circumscribed, of moderate cellularity, composed of bland spindle or plump cells dispersed in a loose-textured and myxoid stroma (Fig. 2). The cytoplasm was eosionophilic or amphophilic without cross-striations. The nuclei were round or elongated, with dispersed chromatin and inconspicuous nucleoli. Multinucleate histiocytoid cells with an eosinophilic or vacuolated cytoplasm were also seen (Fig. 2). The tumor cells were intermingled with an inflammatory infiltrate consisting of plasmocytes, small lymphocytes and occasional eosinophils. Nuclear atypia, mitoses and foci of necrosis were not identified. Immunohistochemically, the tumor cells were consistent with a myofibroblastic phenotype; they expressed strongly vimentin (1 : 100; Dako, Glostrup, Denmark) and smooth muscle actin (1 : 100; Dako) (Fig. 3). Cytokeratin (1 : 75; Dako) and PS100 (1 : 50; Dako) were negative. There was no expression of anaplastic lymphoma kinase ALK (1 : 50; Dako) protein. A marked overexpression of protein p53 (1 : 25; Dako) by tumor cells was found (Fig. 4). Special stains for mycobacteria and fungi were negative. In situ hybridization (PNA probes, Dako) performed to detect Epstein-Barr virus (EBV) was negative. Investigation of Human Herpesvirus-8 (HHV8) by polymerase chain reaction (PCR) was also negative. The histological diagnosis was of an inflammatory myofibroblastic tumor of the retro peritoneum.

## Discussion

Inflammatory myofibroblastic tumor (IMT) is a subtype of inflammatory pseudotumor that is characterized by a proliferation of cells presenting a myofibroblastic differentiation. It is a rare lesion that has been reported with an equal frequency in men and women. It can occur at any age but most often before the age of 40.

IMT occurs commonly in the lung. Extrapulmonary locations are: the liver, the genitor-urinary tract, mesentery, omentum, extremities, head and neck [1-3]. Retroperitoneal sites of involvement are rare. Only 7 cases have been reported in the English literature [1]. The etiopathology of IMT is actually controversial: it is considered by some authors [4] as a reactive inflammatory lesion secondary to surgery, trauma or infection. In the latter, it can be bacterian (*Actinomyces, C. Jejuni or E. Coli*) or viral (EBV or HHV8). Other authors [5] consider IMT like a true neoplasm and even an authentic low grade sarcoma in some cases.

The clinical presentation depends on the location of the tumor. Retroperitoneal tumors usually present with flank or abdominal pain, systemic symptoms such as fever, weight loss and laboratory abnormalities (hypochromic microcytic anemia and raised erythrocyte sedimentation rate); hematuria is also reported [1].

Computerized tomography scan and/or magnetic resonance imaging of IMT usually show a well defined mass. Macroscopically, it is a well circumscribed mass that can be of different colors: yellow, brown or white, depending on the cellular composition of the tumor.

Histologically, IMT corresponds to a fascicular proliferation of spindle cells (fibroblasts, myofibroblasts), intermingled with a chronic inflammatory infiltrate (plasmocytes, lymphocytes, histiocytes and some eosinophilia) and a fibrous connective tissue. There is neither nuclear atypia nor mitoses. Immunohistochemically, spindle cells in IMT show a myofibroblastic immunophenotype, they express vimentin, smooth muscle actin and rarely desmin. Myogenin, myoglobin, CD117 and PS100 are all negative. Actually, cytogenetic and molecular studies tend to consider IMT as a tumor of uncertain malignant potential. In fact, anaplastic lymphoma kinase (ALK) gene abnormalities have been reported in 40% of IMT [1]. Overexpression of p53 that was marked in our case has been reported in recurrent cases or malignant transformation [6]. The histopathological differential diagnosis includes: (1) Malignant histiocytofibroma where tumoral cells are pleomorphic with atypical nuclei, frequent mitoses; (2) Inflammatory fibrosarcoma where nuclear atypia is pronounced (some authors suggest that aggressive IMT with cytogenetic alterations are in fact inflammatory fibrosarcomas); (3) Extramedullary plasmocytoma is a neoplastic proliferation of pleomorphic plasmocytes with numerous mitoses, a thin stroma and a monoclonal expression of immunoglobulin light chains.

The majority of IMT are treated by complete surgical resection. In cases of incomplete resection, a complementary treatment based on corticotherapy can be proposed. Prognosis is excellent when the tumor is completely removed. Recurrence has been reported in 15 to 37% of abdominal IMT [1]. An aggressive behavior has been reported in the head sinuses, the mediastinum and the abdomen; this can be explained by the large size of the tumor or by surgical inaccessibility to the tumor site [7]. Metachronous metastases have also been reported, they are considered by some authors to be a synonym of a malignant transformation of IMT to a low grade inflammatory fibrosarcoma.

In conclusion, inflammatory myofibroblastic tumors are rare lesions of still controversial etiology with an uncertain biological potential that can range from a frequently benign course to a more aggressive evolution. 
